# A Review of Potential Impacts of Climate Change on Coffee Cultivation and Mycotoxigenic Fungi

**DOI:** 10.3390/microorganisms8101625

**Published:** 2020-10-21

**Authors:** Mira Adhikari, Elizabeth L. Isaac, R. Russell M. Paterson, Mark A. Maslin

**Affiliations:** 1 Department of Geography, University College London, London WC1E 6BT, UK; zcfaeis@ucl.ac.uk (E.L.I.); m.maslin@ucl.ac.uk (M.A.M.); 2 Institute for Biotechnology and Bioengineering, Centre of Biological Engineering, University of Minho, 4700-057 Braga, Portugal; russell.paterson@deb.uminho.pt

**Keywords:** coffee, mycotoxins, climate change, species distribution modelling

## Abstract

Coffee is one of the most traded commodities in the world. It plays a significant role in the global economy, employing over 125 million people. However, it is possible that this vital crop is threatened by changing climate conditions and fungal infections. This paper reviews how suitable areas for coffee cultivation and the toxigenic fungi species of *Aspergillus, Penicillium,* and *Fusarium* will be affected due to climate change. By combining climate models with species distribution models, a number of studies have investigated the future distribution of coffee cultivation. Studies predict that suitable coffee cultivation area could drop by ~50% under representation concentration pathway (RCP) 6.0 by 2050 for both Arabica and Robusta. These findings agree with other studies which also see an altitudinal migration of suitable cultivation areas to cooler regions, but limited scope for latitudinal migration owing to coffee’s inability to tolerate seasonal temperature changes. Increased temperatures will see an overall increase in mycotoxin production such as aflatoxins, particularly in mycotoxigenic fungi (e.g., *Aspergillus flavus*) more suited to higher temperatures. Arabica and Robusta’s limited ability to relocate means both species will be grown in less suitable climates, increasing plant stress and making coffee more susceptible to fungal infection and mycotoxins. Information regarding climate change parameters with respect to mycotoxin concentrations in real coffee samples is provided and how the changed climate affects mycotoxins in non-coffee systems is discussed. In a few areas where relocating farms is possible, mycotoxin contamination may decrease due to the “parasites lost” phenomenon. More research is needed to include the effect of mycotoxins on coffee under various climate change scenarios, as currently there is a significant knowledge gap, and only generalisations can be made. Future modelling of coffee cultivation, which includes the influence of atmospheric carbon dioxide fertilisation and forest management, is also required; however, all indications show that climate change will have an extremely negative effect on future coffee production worldwide in terms of both a loss of suitable cultivation areas and an increase in mycotoxin contamination.

## 1. Introduction

The past century has seen unprecedented rates of climate change, with an average warming of 1 °C since the beginning of the industrial revolution [1]. In the 2014 report, The Intergovernmental Panel on Climate Change (IPCC) concluded that, by the end of the century, global average temperatures will be between 1.8 °C and 4 °C higher than preindustrial [1]. Precipitation changes are also expected to be widespread, with an intensification of the water cycle [1] and shifts in rainfall patterns. Climate change is, therefore, going to have a critical effect on the agricultural sector by altering the climatic conditions for plant and mycotoxigenic fungal growth.

Coffee is the world’s 121st most traded agricultural commodity, with over 9.5 billion kg produced in 2018 with a total trade value of $30.9 billion [2]. In Burundi, 17% of the country’s exports are coffee [3] and, in Ethiopia, coffee exports are responsible for 25% of export income [4], highlighting these nation’s dependence on it for economic stability. While countries such as in Brazil and Vietnam produce coffee on an intensive scale [5], in many others such as Ethiopia and Uganda, coffee is grown on small family farms. In Uganda alone, there are around 500,000 farms smaller than one hectare [6]. This means that many farmers do not have access to the funds and infrastructure for irrigation or expensive pesticides/fungicides, relying entirely on the natural climate to grow their crops. This makes them particularly vulnerable to mycotoxin contamination. Coffee production increased by 57% over the period 2012–2016 [7]. This reflects the rising demand and the growth of this part of the agricultural sector; therefore, the importance of investigating how climate change will affect the plant cannot be overlooked. The increased demand for coffee also highlights the need to understand how mycotoxin concentrations will change in the future to be able to effectively regulate safe limits for human consumption. This paper reviews the current state of research on the impact of climate change on coffee production which will be highly associated with mycotoxigenic fungi growth and mycotoxin production [8].

## 2. Coffee Growth Cycle

Germination of a coffee seed takes 1–2 months [9] and the plant takes approximately 3 years to mature and begin producing fruit [10]. The growth of flower buds is stimulated by day length and cooler temperatures, conditions that are present as the dry season begins [10]. During the dry season, flower buds become dormant and flowering can only begin once this dry spell is broken [9]. From here, the plant is fertilised either via self-pollination (pollination from the same tree) for Arabica or cross-pollination (pollination from a different tree) for Robusta [10]. The flowers bloom for less than two weeks, followed by the development of the coffee cherries. Development takes 6–9 months for Arabica and 9–11 months for Robusta [9]. At this stage, the berries turn from green to deep red and are ready for harvesting. Cultivated coffee trees have a life span of ~30 years [11]. Although they can live up to 80 years in the wild, the most productive years are 7–20.

## 3. Optimal Climatic Conditions for Coffee

Most coffee grown is made up of two species: *Coffea arabica* (Arabica) and *Coffea canephora* var. Robusta (Robusta), with the former making up 70% of all coffee grown globally, and the latter encompassing the remaining 30% [3]. Temperature and rainfall are important variables as the onset of the dry season and drops in temperature are key in terms of the plant flowering and, thus, producing beans. As coffee species do not tolerate large changes in seasonality, yet do not flourish in very hot conditions, altitude is also an important factor. For every 100 m, temperature decreases on average by 0.6 °C [12]. Higher altitudes in tropical regions, therefore, offer a cool environment where the yearly temperature does not fluctuate drastically, providing an ideal climate for coffee cultivation. The altitude cannot be too high, however, as temperatures below freezing can lead to frost damage, an irreversible process where ice forms within the cells, expanding them and causing the walls to burst [9].

Arabica coffee originated in Ethiopia, growing within tropical forests at altitudes of 1600–2800 m [13]. This region has well-distributed rainfall patterns, ranging from 1400–2000 mm for two-thirds of the year, with the last third being the dry season and coinciding with the coldest months [14]. Arabica’s optimum rainfall is between 1200 and 1800 mm annually, and it prefers temperatures between 18 and 22 °C with little seasonal fluctuations [15], although it can tolerate temperatures as low as 15 °C and up to 25 °C [9]. However, at these higher temperatures, photosynthesis is reduced, and chlorosis (yellowing of the leaves due to damaged chlorophyll) can occur [9]. These environmental requirements mean that Arabica is mostly grown in regions around the equator, with the major production areas being the Brazilian highlands, Central America, and Colombia [16].

Robusta is native to forests at lower altitudes surrounding the Congo river basin [15], below 1000 m. The rainfall in this area is around 2000 mm, falling over a 10 month period, making the dry season much shorter [13]. Robusta’s optimum rainfall is between 2000 and 2500 mm [9]. Although it is more resistant to higher temperatures than Arabica (optimum temperatures being between 22 and 28 °C), it is more sensitive to lower temperatures [11]. Photosynthesis becomes reduced only after temperatures over 30 °C are reached [9]. Robusta becomes vulnerable when the dry season exceeds 3–4 months due to high evapotranspiration. This is because of the higher temperatures it grows in and its preference for a higher relative humidity than Arabica (75% vs. 60%) [9]. Robusta’s major exporters are Vietnam, West Africa, and Indonesia [16].

Coffee species are vulnerable to a wide range of pests, bacterial, and fungal diseases. The biggest pest threat to coffee species is the coffee berry borer (*Hypothenemus hampei*), an insect which burrows into the coffee fruit, laying its eggs inside. When the larvae hatch, they consume what is left of the berry, turning it rotten [17]. The insect damage is known to cause an increase in fungal infection [8]. Bacterial diseases include halo blight, leaf spot, and coffee leaf scorch and are responsible for significant reductions in coffee yields [18]. Fungal diseases include coffee berry disease, coffee wilt disease, and coffee leaf rust.

## 4. Mycotoxigenic Fungi

This review focuses on the mycotoxins which are produced by some species of fungi. Mycotoxins are secondary metabolites produced by toxic fungi that have infected the plant either during cultivation or after (during processing or transport) and, thus can contaminate coffee at almost any stage [19]. These mycotoxins can cause sickness or death if ingested or inhaled by humans or animals [8]. The major mycotoxin-producing fungal species are *Aspergillus, Penicillium*, and *Fusarium*, which produce mycotoxins such as ochratoxin A (OTA), fumonisins, and aflatoxins [8,20].

Ochratoxin A (OTA) is produced by several *Aspergillus* species, including *A. ochraceus, A. alliaceus, A. carbonarius, A. glaucus*, and *A. niger* [19]. It is also produced by *Penicillium verrucosum*. In tropical conditions where coffee is grown, *A. ochraceus* is the major source of OTA, with optimum temperatures for production being between 25 and 30 °C [21]. *P. verrucosum* grows optimally at lower temperatures (26 °C) and produces OTA at 25 °C [8]. Clearly, the ambient temperature of where coffee is grown will affect the growth of these fungi. This mycotoxin has been found on a range of crops, including barley, grapes, rye, wheat, and coffee. OTA is a kidney toxin, carcinogen, and immunosuppressant [22]. Table 1 indicates the concentrations of OTA in various countries around the world and the global mean concentration.

Fumonisins are a group of 15 mycotoxins documented to have contaminated a range of different crops from maize and grains, to vines, although the major mycotoxin is fumonisin B_1_ [24]. Fumonisins are predominantly produced by *Fusarium verticillioides*, *Fusarium proliferatum*, and *Fusarium nygamai* [19]. They are linked with oesophageal cancer and have carcinogenic effects on animals [19]. Fumonisin production is thought to reach its maximum at around 30 °C [25,26].

Aflatoxins are the most toxic of all of the mycotoxins covered in this review and have one of the higher recorded temperature optima (33–35 °C) of the three mycotoxins, meaning that they may persist in the most extreme climate warming conditions [27]. They also prefer higher humidity, making them well suited to tropical conditions. Aflatoxins are produced predominantly by *Aspergillus flavus* and *Aspergillus parasiticus* [8]. The dominant aflatoxin produced (aflatoxin B_1_) is also the most powerful naturally occurring carcinogen [19] and, hence, it is of particular importance to understand how levels of this mycotoxin may shift with the climate change that coffee will also experience. Aflatoxin contamination is worst during drought years as they are xerotolerant [19], and production reaches its optimum between 28 and 30 °C [28], while *A. flavus* itself can tolerate temperatures from 19–35 °C.

As mycotoxins can have such harmful consequences on human health, they are regulated by various bodies such as the Food and Drug Administration (FDA) and European Union (EU). Safe limits are usually based on acceptable daily quantities of each specific mycotoxin, although regulations vary between different institutions and there are no universal guidelines [19]. Monitoring mycotoxin levels in crops is critical as failure to do so can lead to large amounts of contaminated produce being destroyed, resulting in profound economic losses [19] or serious risk to people. Crops containing mycotoxins can also sometimes be processed into animal feed, which, once ingested, can accumulate into milk or animal tissue [29,30] that is then consumed by humans. Good farming practice such as proper drying of crops can help to prevent mycotoxin contamination post harvest; however, there are still challenges with regard to preventing preharvest contamination [19].

## 5. Climate Change Impacts on Coffee Cultivation Regions

Most studies to date that explored climate change’s impact on coffee cultivation have been region- or country-specific and have focused solely on Arabica. Baca et al. [31] used niche modelling to investigate Arabica growth in Mesoamerica and found a significant decrease in climate suitability at low altitudes. At present, the most suitable elevation for growing in this region is 1200 m but the study found that this will increase to 1600 m by 2050. Similarly, in Sierre Madre, Mexico, Arabica suitability will show a strong decline by 2050, particularly below 1700 m where the majority is currently farmed [32]. In Brazil, Zullo et al. [33] mapped risk areas and found that, under rising mean temperatures and reduced frost risk, Arabica cultivation could migrate latitudinally to more southern states.

Moat et al. [34] used an ensemble SDM (species distribution model) method to look at Ethiopia’s suitability for Arabica and found that, without intervention, by 2099, large declines in the suitable area available (between 39% and 59%) would be seen. However, they also found that a combination of forest management and a relocation of growth areas to higher altitudes could lead to a 400% increase in suitable area, but this would require extensive intervention such as the planting of shade trees. A decrease in suitable area was also found in Uganda by Jassogne et al. [35], rendering areas at elevations less than 1300 m completely unsuitable for Arabica. Davis et al. [36] used MaxEnt, a learning algorithm often used for SDM to explore how climate change will affect indigenous Arabica. They found, for their locality (area) analysis, even in the best case scenario, there would be a 65% (38%) reduction in present-day suitable land within Africa (predominantly in Ethiopia, Africa’s leading Arabica exporter) by 2080, with the worst case scenario resulting in a 100% (90%) loss of land.

Vietnam, the second largest exporter of coffee, is expected to undergo substantial losses, with any gains in suitability often being located in protected areas that are not open to farming [37]. Elsewhere in Asia, Indonesia, another major exporting country, is anticipated to lose up to 90% of its suitable land in provinces such as North Sumatra and Aceh [38]. Additionally, Schroth et al. [38] found an altitudinal migration of suitable areas in Indonesia which was also seen in Schroth et al.’s [32] findings for Mexico.

Haggar and Schepp [14] extended their analysis to multiple countries: Brazil, Vietnam, Guatemala, and Tanzania. All four of these countries are expected to undergo overall losses of suitable land under multiple climate change scenarios, although some regions such as the northern Sierras in Guatemala and Rio Grande do Sul in Brazil will increase in suitability.

**Figure 1:**
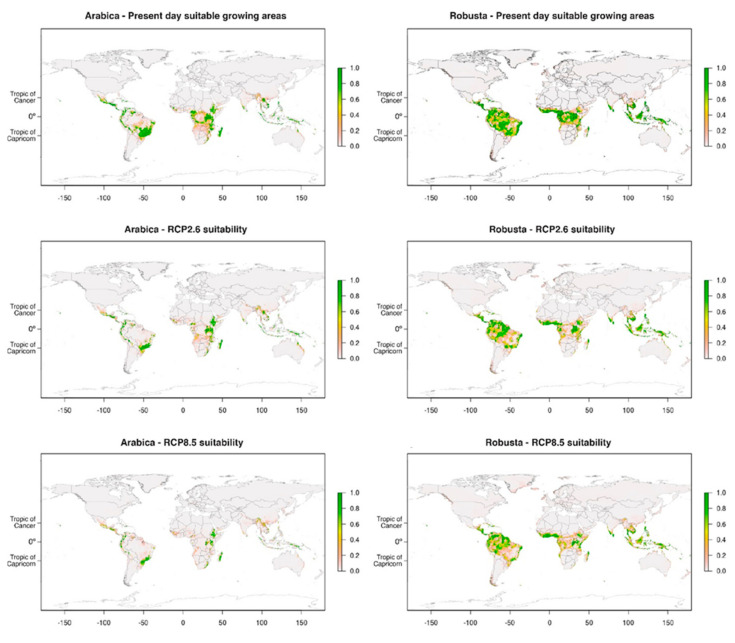
Present-day and future suitability maps for Arabica and Robusta. A value of 1 indicates a high probability (certainty) of species presence in the region, while a value of 0 indicates a very low probability of species presence on the basis of bioclimate conditions. Future scenarios presented are those of the Intergovernmental Panel on Climate Change (IPCC) (2013) RCP 2.6 and 8.5 for 2070. Source: Adhikari et al. [41].

To date, the only global study has been performed by Bunn et al. [11] using three machine learning algorithms: MaxEnt, random forest, and support vector machines. The algorithms were used in conjunction with five general circulation models and the 19 bioclimatic variables to investigate present and future distributions of both Arabica and Robusta. Here, as in other studies [34,39] temperature variables were more important than precipitation ones, although precipitation appeared slightly more important for Robusta than Arabica. Like Zullo et al. [33], Bunn et al. also saw a southern migration of suitable area for Brazil, as well as a general trend of altitudinal migration globally. Vietnam will lose considerable area by 2050, as will regions in West Africa. Brazil and southeast Asia will show the biggest declines in suitable area for Arabica, whilst the Congo will have the biggest declines for Robusta. In general, the greatest losses were seen at low altitudes, as well as in areas without forest cover, further highlighting the potential importance of forest management as a tool to cope with climate change as mentioned in Moat et al. [34] and Magrach and Ghazoul [40]. Overall, land losses of 50% are projected to occur under RCP scenario 6.0 by 2050 for both species, with slight gains in East Africa and the Asian Island States (Figure 1).

These results are very similar to those found by Adhikari et al. [41], who used boosted regression trees to perform a similar species distribution modelling study. As with Bunn et al. [11], the major drivers of coffee distribution were identified as temperature seasonality and the annual temperature range. Adhikari et al. found that, by 2070, Robusta could lose ~25% of its suitable area under RCP 2.6, 4.5 and 6.0 (Figure 1). Further losses only occur under RCP 8.0 with a loss of ~35%. For Arabica, the losses will be more severe, with losses of ~40% for RCP 2.6 and 4.6, and ~95% for RCP 6.0 and 8.5.

Bunn et al.’s [11] study found that Robusta will undergo more severe losses than Arabica. This contradicts the idea that Robusta is more heat-tolerant and, therefore, better suited to increasing temperatures (e.g., Magrach and Ghazoul [40]). The general consensus that Robusta’s optimal temperature range is between 22 and 28 °C [9] comes from the climatic conditions of its native habitat. This was challenged by Kath et al. [42]. Using temperature and precipitation data and yields across southeast Asia, the authors used hierarchical Bayesian modelling to investigate Robusta’s optimal temperature and found that it lies below 20.5 °C, which is significantly lower than previously thought. Robusta’s optimal temperature was previously thought to be above 25 °C; however, the authors saw that yields were 50% lower at this temperature compared with yields at 20.5 °C. This suggests that previous studies may have overestimated Robusta’s ability to tolerate climatic change, as well as its ability to replace Arabica as temperatures increase.

Many studies also overlooked the role that increasing CO_2_ concentrations may have on coffee growth. Those that investigated this found that increasing CO_2_ concentrations make coffee species more resilient to increasing temperatures by enhancing photosynthetic processes and potentially even improving crop yields [43,44,45]. Further work is, therefore, needed to include CO_2_ in the future as it may be that previous research underestimated coffee’s resilience in a higher-CO_2_ world [46].

## 6. Climate Change Impact on Mycotoxins and Mycotoxigenic Fungi

Currently, there is a significant lack of research with regard to climate change impacts on mycotoxigenic fungi on coffee; however, general patterns can be discussed on the basis of previous research for other crops and on environmental studies on the fungi and mycotoxins themselves.

As OTA is optimally produced by *A. ochraceus* between 25 and 30 °C, but only at 25 °C for *P. verrucosum* [21], it is likely that the latter will become less prominent in OTA production due to climate change. Climatic change will not only involve increases in temperature, but also an increase in intensity of the water cycle [2]. As coffee already requires wet conditions, the rainfall and humidity in cultivation areas is likely to increase, and, as *Aspergillus* species prefer hotter and more humid climates [47], they will likely thrive in these conditions. Studies conducted on OTA and wine have shown that elevated CO_2_ concentrations, temperature, and humidity all result in increased OTA production [24,48].

*A. flavus* can grow in a wide range of temperatures (between 19 and 35 °C) and, thus, it is well suited to cope with future climatic shifts. Its optimum temperature for aflatoxin production is between 28 and 33 °C [21]. This is higher than for OTA production by *A. ochraceus*, which could mean that, in the future, aflatoxins may become the most dominant mycotoxin. This is an area for concern as aflatoxins are classed as group 1 carcinogens [24]. Increases in this mycotoxin have already been shown for maize in modelling studies for Europe [49].

Fumonisin production is thought to reach its maximum at around 30 °C [25,26] and, hence, this mycotoxin is also likely to increase with climate change. Although *Fusarium* species are also tolerant to higher temperatures, they are not as xerotolerant as *A. flavus* [50]; thus, in areas where rainfall is predicted to decrease, fumonisins may be replaced by aflatoxins. However, as coffee has higher rainfall requirements, drought is unlikely to be an issue and *Fusarium* species are likely to continue to thrive under climate change with increasing temperatures.

Overall, there is an apparent likelihood that, in an increasingly warmer world, mycotoxin production will rise, as higher temperatures and wetter climates provide ideal conditions for fungi and, thus, mycotoxin production [51]. Climate change will also bring about shifts in which mycotoxins are most prevalent, for example, *Penicillium* species may decline, and there may be an increase in aflatoxin-producing *Aspergilli* species, which are more hazardous to human and animal health.

Additionally, climate change will indirectly increase fungal growth on coffee due to plant stress. As coffee has strict environmental requirements and limited space for altitudinal migration as seen in the previous section, it is likely that current cultivation areas will become less bioclimatically suitable. The resultant stress on the coffee plants can lead to them becoming more prone to fungal infection and mycotoxin contamination [27]. The susceptibility to fungal infection has been determined for oil palm, where Paterson [52,53] modelled the effect of future climate on the incidence of the devastating fungal disease of basal stem rot caused by *Ganoderma boninense*. The results showed that decreasing climate suitability reduced the plant’s resistance to the disease significantly, and increased mortality of oil palm was also predicted. The species distribution modelling studies showed that some land at higher altitudes will become more bioclimatically suitable for coffee cultivation in future, and, if coffee plantations are shifted to these areas, farms may benefit from a “parasites lost” phenomenon, where they are less prone to infection as they have left the fungus behind [27]. Robusta is likely to fare better than Arabica as it can be grown in a wider range of conditions and is a more tolerant species. This means that it will not only fare better against changes in climate, but also be less likely to suffer stress and be more resistant to infection.

Finally, as discussed in the previous section, an increase in CO_2_ may benefit both species of coffee and improve yield; however, these studies did not consider the fact that increased CO_2_ concentrations may also affect mycotoxigenic fungi. While little research exists for coffee, studies have found that both maize and wheat become more susceptible to *Fusarium*-related diseases with increased CO_2_ concentrations, although whether this means there will be an increase in fumonisins is less clear [54,55].

Ghini et al. [56] provided the first evidence that the fungal community associated with mycotoxins may not be affected by the CO_2_ treatments in free-air CO_2_ enrichment undertaken in Brazil. The authors mentioned that special care must be exercised when analysing these data as they represent the first harvest, and the elapsed time under elevated CO_2_ might not have been sufficiently long to affect the population of fungal production. However, Akbar et al. [57] stated that there was only a significant stimulation of OTA production by *A. westerdijkiae* strains in elevated CO_2_ (1000 ppm) at 0.90 a_w_ on stored coffee beans. Akbar et al. [58] demonstrated a high OTA contamination of stored green coffee when colonised by *A. westerdijkiae* strains, especially at 0.95 and 0.97 a_w_ and 30 °C, regardless of CO_2_ levels. OTA contamination levels were significantly lower than at 35 °C than 30 °C on every occasion. However, there were higher OTA contamination levels from strains at 0.95 and 0.90 a_w_ and 1000 ppm CO_2_ at 35 °C. These results suggest that the stress of interacting abiotic factors results in a stimulation of OTA production by this species, which is relevant to climate change. The differences between the type strain, *A. westerdijkiae* (CBS 121986), and all the other strains employed were interesting and it would be useful to know why the strains diverged.

## 7. Current Observations

Within the agricultural sector, changes in mycotoxigenic fungi are already being seen due to climate change. Dry and hot weather in Europe contributed to a 2003 outbreak of *A. flavus* on crops in Italy, uncommon previously [51]. *A. flavus* was able to colonise ripening maize by outcompeting the more common *Fusarium* species, causing an increase in aflatoxin B1 contamination. Furthermore, increased notifications of aflatoxin M1 in milk in 2012 were related to an increase of aflatoxins in maize for animal feed from southeast Europe. The maize survived a severe drought, making the crop vulnerable to infection by *A. flavus*/*A. parasiticus*, resulting in increased aflatoxins. A large survey established aflatoxin contamination of corn, almonds, and pistachios grown in southern Europe due to the recent development of a subtropical climate. Furthermore, a model developed for *A. flavus* growth and aflatoxin B1 production indicated a clear increase in aflatoxin risk in areas in central/southern Spain, south Italy, Greece, north/southeast Portugal, Bulgaria, Albania, Cyprus, and Turkey with an increase of +2 °C. *A. flavus* was able to colonise maize at the ripening stage by outcompeting *Fusarium* species. Drought and extreme elevated temperatures (>35 °C) resulted in a change from *Fusarium verticillioides* and contamination with fumonisins of maize to *A. flavus* and aflatoxins. Reduced sporulation occurred at dry conditions of 0.90 water activity (a_w_), but *A. flavus* can even grow at 0.73 a_w_ and produce aflatoxins at 0.85 a_w_, while *F. verticillioides* growth is low at 0.90 a_w_ and fumonisins are produced only at >0.93 a_w_ [51]. Furthermore, aflatoxin contamination was not detected initially in Serbian maize, but hot and dry weather resulted in 69% of samples being contaminated with aflatoxins. Mycotoxin levels in cereal and mixed feed samples collected in Hungary were examined, and aflatoxin B1 levels above the EU limit were observed in 4.8% of the samples. Aflatoxin in maize (2003) and milk (2007, 2011, 2012, 2013) originating from Hungary, Serbia, Romania, and Slovenia was detected within the framework of the Rapid Alert System for Food and Feed of the European Union. Due to the extreme weather conditions in 2012 in Central Europe, aflatoxin contamination of maize and milk caused serious problems in Serbia, Romania, and Croatia. Aflatoxins were also detected in maize kernels in Hungary after harvest in 2012. High temperatures favour the growth of the fumonisin producer *F. verticillioides* in maize [51]. Displacement of the formerly predominant species, *F. culmorum* and *Microdochium nivale*, by the more virulent plant pathogen *F. graminearum* as a result of warm European summers has been reported. A more toxigenic 3-acetylated deoxynivalenol (3ADON) chemotype of *F. graminearum* replaced the 15-acetylated deoxynivalenol (15ADON) chemotype in Canada. In contrast, Minnesota, the United States of America (USA) has witnessed the emergence of a novel *Fusarium* isolate called the “Northland population”, which does not produce the trichothecenes deoxynivalenol or nivalenol [51], partially confirming that it is conceivable that reduced mycotoxins may occur with climate change.

Increasing temperatures have resulted in the coffee berry borer being found at elevations 300 m higher 10 years ago in the Kilimanjaro region [59]. In Ethiopia, the insect is now also able to complete more generations per year [60]. Not only will the increase in pests themselves bring more mycotoxigenic fungi, it will also render coffee plants more vulnerable to disease by destroying them. East African coffee farmers have also described changes in annual weather patterns, with longer dry seasons and heavier rainfall in the wet season [39]. This has had negative effects on the flowering process and, thus, the timing of cherry initialisation. Veracruz, Mexico received 40 mm less rainfall per year from 1969–1998 [61]. Coffee yields in Costa Rica have declined as mean and maximum temperatures rise [62]. In Tanzania, declines in Arabica were shown to be most affected by increasing night-time temperatures between 1961 and 2012 [39]. This evidence that changes are already underway highlights the pressing need to further understand and mitigate their consequences.

## 8. Conclusions

In summary, both Arabica and Robusta will see declines in important cultivation areas such as Brazil, Vietnam, and Ethiopia due to climate change. Suitable areas will migrate to higher altitudes where temperatures are cooler; however, latitudinal shifts are constrained by both species’ need for little fluctuation in temperature. Generally, Arabica fares worse than Robusta (although there are exceptions as shown by Bunn et al., 2015), with Robusta actually gaining suitable area in some future scenarios.

Increased temperatures will also see an overall increase in mycotoxin production, particularly for those more suited to higher temperatures such as aflatoxins, which is of particular concern due to the fact they are a class I carcinogen. Arabica and Robusta’s limited ability to relocate means that both species will be grown in less suitable climates, increasing plant stress and making coffee more susceptible to fungal infection and mycotoxins contamination. However, in a few areas where relocating farms is possible, mycotoxin contamination may decrease due to the “parasites lost” phenomenon. More research is needed to understand how shifts in suitable areas for Arabica and Robusta will affect the fungi and their mycotoxins under various climate change scenarios. Further studies should also focus on the impact that increasing CO_2_ growth will have on both coffee and mycotoxigenic fungi. Considering coffee’s importance as a cash crop, there are currently significant gaps in the knowledge when compared with other crops such as maize or wine.

## Figures and Tables

**Table 1:** Ranked order of countries on the basis of mean concentration of ochratoxin A (OTA; mg/kg) in coffee and coffee-based products (Khaneghah et al. 2019 [23]).

Country/Territory	OTA Concentration (mg/kg)	Country/Territory	OTA Concentration (mg/kg)
Turkey	79.0	Brazil	1.81
Philippines	52.7	Portugal	1.71
France	38.9	Ethiopia	1.53
Panama	21.3	Argentina	1.39
South Korea	5.62	Switzerland	1.30
Spain	4.52	Italy	1.21
Cyprus	3.90	Chile	1.10
Malaysia	3.48	Thailand	0.89
Vietnam	2.86	Czech Republic	0.84
Denmark	2.82	Japan	0.48
Kuwait	2.56	Taiwan	0.35
		Global pooled mean	3.21
